# Unsaturated Fatty Acids Complex Regulates Inflammatory Cytokine Production through the Hyaluronic Acid Pathway

**DOI:** 10.3390/molecules28083554

**Published:** 2023-04-18

**Authors:** Gi-Beum Kim, Kwansung Seo, Jong-Ung Youn, Il Keun Kwon, Jinny Park, Kwang-Hyun Park, Jong-Suk Kim

**Affiliations:** 1 Department of Biochemistry and Molecular Biology, Jeonbuk National University Medical School, Jeonju 54907, Republic of Korea; 2 Department of Dental Materials, School of Dentistry, Kyung Hee University, Seoul 02447, Republic of Korea; 3 Eouidang Agricultural Company, Wanju, Jeonbuk 55360, Republic of Korea; 4 Division of Hematology, Gacheon University Gil Medical Center, Incheon 21565, Republic of Korea; 5 Department of Emergency Medicine and BioMedical Science Graduate Program (BMSGP), Chonnam National University, Gwangju 61469, Republic of Korea

**Keywords:** plant-derived unsaturated fatty acid, antioxidant, anti-inflammatory, hyaluronic acid, hyaluronic acid synthase

## Abstract

In this study, we aimed to develop natural and/or functional materials with antioxidant and anti-inflammatory effects. We obtained extracts from natural plants through an oil and hot-water extraction process and prepared an extract composite of an effective unsaturated fatty acid complex (EUFOC). Furthermore, the antioxidant effect of the extract complex was evaluated, and the anti-inflammatory effect was explored by assessing its inhibitory effect on nitric oxide production through its HA-promoting effect. We conducted a 3-(4,5-dimethylthiazol-2-yl)-2,5-diphenyl-2H-tetrazolium bromide assay to evaluate the cell viability of the EUFOC, and the results showed that EUFOC was not cytotoxic at the test concentrations. In addition, it showed no endogenous cytotoxicity in HaCaT (human keratinocyte) cells. The EUFOC showed excellent 1,1-diphenyl-2-picrylhydrazyl- and superoxide-scavenging abilities. Moreover, it exerted an inhibitory effect on NO production at concentrations that did not inhibit cell viability. The secretion of all the cytokines was increased by lipopolysaccharide (LPS) treatment; however, this was inhibited by the EUFOC in a concentration-dependent manner. In addition, hyaluronic acid content was markedly increased by the EUFOC in a dose-dependent manner. These results suggest that the EUFOC has excellent anti-inflammatory and antioxidant properties, and hence, it can be used as a functional material in various fields.

## 1. Introduction

Different health problems have been attributed to environmental pollution and climate change [1,2]. In addition, the healthcare and anti-aging industries are growing rapidly in response to economic growth and cultural influence, and the development of new materials and additional functional exploration of existing materials are continuously increasing to meet various social demands and consumer needs [3]. Scientific evidence has increased the reliability of the pharmaceutical and healthcare industries, and the search for preventive/healthcare materials from synthetic and natural products and the application of these products have been expanding [4,5].

The interest in general healthcare is expanding to natural materials, and scientific approaches to natural materials that have been traditionally used are also increasing [6,7]. Natural materials are being developed for healthcare in various forms, and the efficacy and safety of these raw materials are recognized as important research targets [8].

Integumentary system management can be defined as the act of restoring or sustaining a human body’s function through various physical stimuli using the hands or devices [9,10]. In other words, unlike in the past, integumentary system management in Korea has moved away from aesthetic concepts and is now focused on the health of the human body, which is a more fundamental and comprehensive concept [10,11]. Many of these alternative therapies have already been introduced at the integumentary system management level and are being performed in new ways.

The stratum corneum, located in the uppermost part of the integument system, acts as a barrier to prevent evaporation and the loss of water molecules, thereby protecting the body from external chemical and physical damages and preventing bacteria, fungi, and viruses [12]. However, if pathogens cause microcracks and crevices in the epidermis, irritants and allergens may invade these areas. The stratum corneum plays an important role in the primary defense line [12].

Therefore, it is essential to adopt an external therapy for the regeneration or maintenance of the stratum corneum barrier. However, external treatment in Korean oriental medicine is mainly used as an adjuvant treatment [12].

The integument protects the body against various stimuli from the external environment and is an important organ for maintaining homeostasis in the human body [13]. Exposure to UV rays not only causes dermal pigmentation and inflammation but also accelerates aging. Macrophages are activated by inflammatory irritation, and they secrete inflammatory factors, including nitric oxide (NO), prostaglandin E2 (PGE2), tumor necrosis factor-α (TNF-α), and interleukins [14,15,16,17,18].

Medicine and healthcare have different applications in dermatology, including skin whitening, wrinkle improvement, and UV protection. Wrinkle improvement is determined through the analysis of indicators, such as water content and dermal density [18]. One of the methods for maintaining and improving tissue hydration in the integument system is the use of hyaluronic acid (HA), which is distributed in the dermal layer as a hydrophilic polymer and is one of the three physical elements of collagen and elastin [19]. HA is closely related to hydration in the dermal system, and water molecules can be preserved by inhibiting hyaluronidase [20]. HA is a glycosaminoglycan that can be synthesized in the human body and is closely related to the maintenance of water molecules in the integument system and human tissues, as it forms a gel by combining with water molecules [21]. It is also essential for maintaining the elasticity and structure of collagen [22]. HA patches and the direct injection method have been developed in the medical and healthcare industries [23,24]. HA is highly hydrophilic, and hence, denaturation can occur easily if exposed to air for a long time or left in a humid environment. Therefore, the necessary technical expertise is required for the manufacturing of the HA patch.

Although the types of alternative medicine are not clearly established in Korea, Korean oriental herbal medicine is the most common type, accounting for 45.9%, and it includes acupuncture, moxibustion, folk remedies, acupressure, spinal correction, and therapeutic touch, indicating that naturopathy and manipulative therapy are the most common alternative medicines [24].

The natural plant materials used in this study were *Helianthus annus* seeds, *Perilla frutescens*, *Prunus armeniaca*, *Paeonia lactiflora*, *Morus mongolica* (Bureau) C. K. Schneid, *Angelica gigas*, *Sophora flavescens*, *Gardenia jasminoides*, *Saururus chinensis*, *Houttuynia cordata*, *Acanthopanax cordata*, *Polygala tenuifolia*, and *Cimicifuga racemosa*. The characteristics of each plant are described in the Supplementary Materials of previously reported studies [25,26,27,28,29,30,31,32,33,34,35,36,37,38,39,40,41,42].

This study aimed to develop healthcare products for the treatment and prevention of inflammation using the aforementioned natural plant materials. We extracted oil components from natural plants, and their applicability as medicinal and healthcare materials was confirmed by verifying their antioxidant and anti-inflammatory effects through the inhibition of NO production and the promotion of HA production.

## 2. Results and Discussion

### 2.1. Endogenous Cytotoxicity of the EUFOC

Staining is the most widely used method to quantify biofilms, and the main staining targets are cells, extracellular polymeric substances (EPS), proteins, and DNA. Fluorescent staining agents are mainly used, but general staining agents with colors, such as crystal violet, are also used. When a staining agent binds to a specific molecule, species, or microorganism in a specific state, staining may also be used for qualitative analysis [43].

Crystal violet staining is the most commonly used method for biofilm staining. It is generally used in static biofilm analysis with a microtiter plate and is an easy, fast, and highly reproducible method. Crystal violet stains both live and dead cells. Because a very mature biofilm is composed of several layers of cells and EPS, which interfere with staining, this method is particularly useful in the early–middle stages of biofilm. Microorganisms were grown in microtiter plates and biofilms formed on the inner wall of the plate wells. Next, the medium was discarded, the suspended microorganisms were washed off and removed, and the attached biofilms were stained with crystal violet. Because the amount of crystal violet reflects the amount of biofilm, the crystal violet was dissolved in ethanol, and the absorbance was measured and quantified at 550 nm. High absorbance indicates the formation of a large amount of biofilm [44].

**Figure 1:**
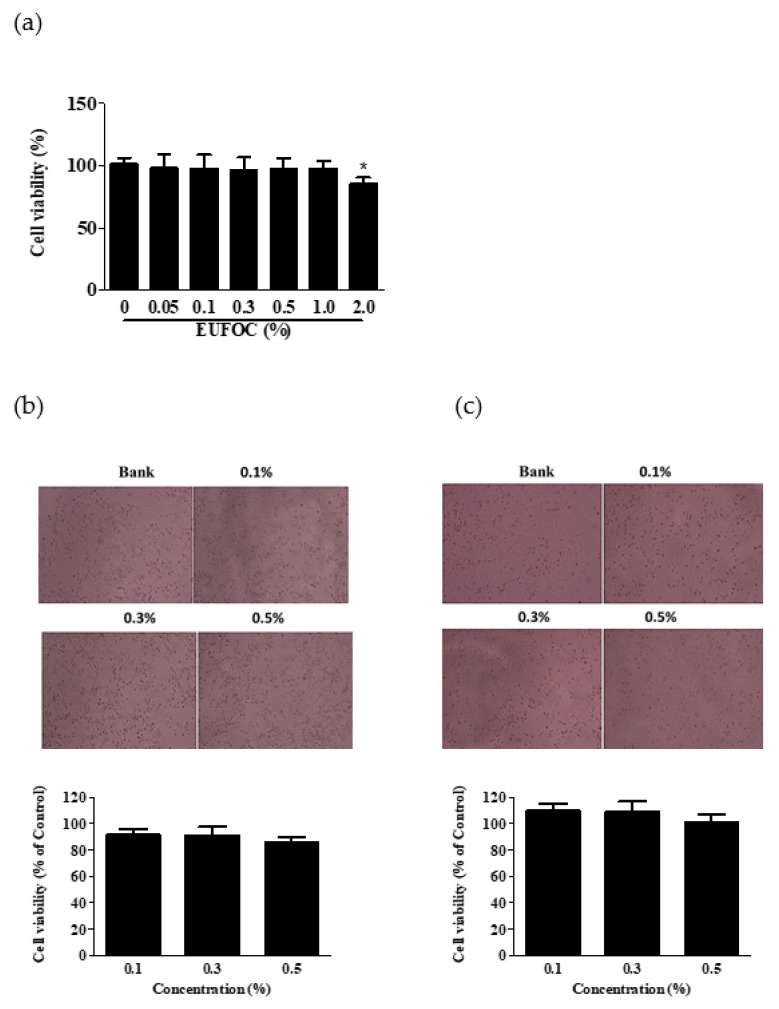
Assessment of cell viability of EUFOC. (**a**) Cell viability was measured using the MTT assay. * *p* < 0.05 vs. negative control group. Endogenous cytotoxicity of EUFOC in (**b**) human embryonic kidney 293T cells and (**c**) HaCaT cells was measured by crystal violet staining. Representative microscopic images (upper panel) and statistics analysis graph (lower panel) are shown in (**b**,**c**). EUFOC: effective unsaturated fatty acids complex; MTT, 3-(4,5-dimethylthiazol-2-yl)-2,5-diphenyl-2H-tetrazolium bromide.

The effect of the EUFOC on cell viability was confirmed by 3-(4,5-dimethylthiazol-2-yl)-2,5-diphenyl-2H-tetrazolium bromide (MTT) assay and crystal violet staining (Figure 1). The results showed that the EUFOC did not exert endogenous cytotoxicity up to a concentration of 1.0% (Figure 1a). In addition, the results of cell viability tests on 293T (Figure 1b) and HaCaT (human keratinocyte) cells showed that the oil was not cytotoxic at concentrations of 0.1, 0.3, and 0.5%. These results showed that the prepared oil was very safe for the cells (Figure 1c).

### 2.2. Evaluation of the Antioxidant Property of the EUFOC

#### 2.2.1. Analysis of 1,1-diphenyl-2-picrylhydrazyl-Scavenging Ability of the EUFOC

**Figure 2:**
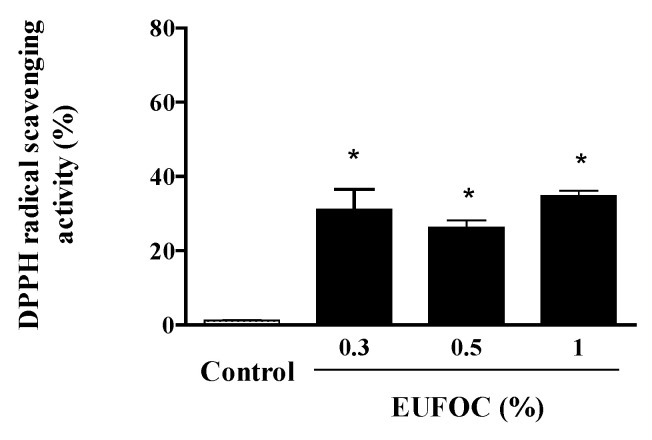
Analysis of DPPH radical-scavenging activity of EUFOC. EUFOC: effective unsaturated fatty acids complex; DPPH, 1,1-diphenyl-2-picrylhydrazyl. Control: negative control (distilled water). * *p* < 0.05 vs. control group.

The 1,1-diphenyl-2-picrylhydrazyl (DPPH) free-radical-scavenging ability of the EUFOC was also analyzed. The cells were treated with 0.3, 0.5, and 1% EUFOC, which were not in the cytotoxic range. The results showed that the scavenging ability, which was 15–35%, increased in a concentration-dependent manner (Figure 2).

#### 2.2.2. Analysis of Superoxide-Scavenging Ability of the EUFOC

**Figure 3:**
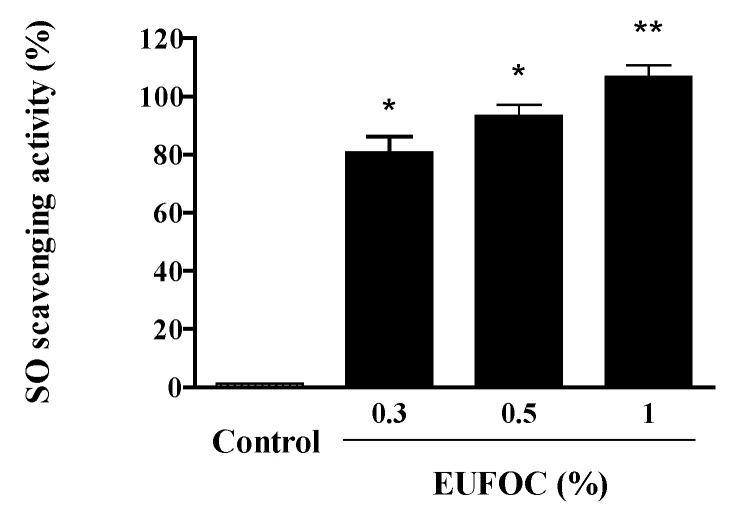
Analysis of the superoxide (SO)-scavenging ability of EUFOC. EUFOC: effective unsaturated fatty acids complex. Control: negative control (distilled water). * *p* < 0.01 and ** *p* < 0.001 vs. control group.

At concentrations of 0.3, 0.5, and 1%, the EUFOC showed a relatively high clearance of >80% in a concentration-dependent manner (Figure 3).

### 2.3. Inhibition of NO Production

Oxygen is converted into a significant amount of reactive oxygen during the oxidation process in the body. It reacts with cellular components in the body, causing oxidative stress that damages cells and tissues in the body [45,46]. Continuous oxidative stress results in DNA denaturation, damages cell membranes and proteins, and induces chronic inflammatory reactions [47,48].

**Figure 4:**
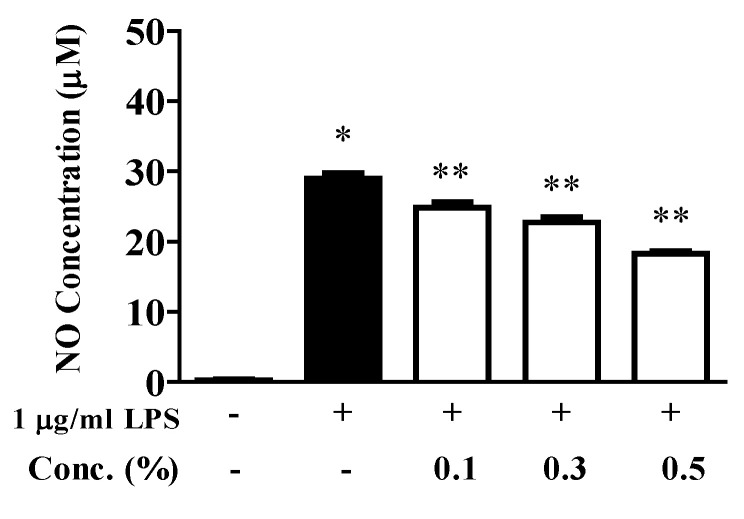
Inhibitory effect of EUFOC on lipopolysaccharide (LPS)-induced nitric oxide production. EUFOC: effective unsaturated fatty acids complex. * *p* < 0.001 vs. negative control group, ** *p* < 0.01 vs. LPS-treated group.

The inhibitory effect of the EUFOC on NO production was exhibited at concentrations that did not inhibit cell viability (Figure 4), indicating the anti-inflammatory potential of the EUFOC.

### 2.4. Inhibitory Effect of the EUFOC on the Production of Inflammatory Cytokines

**Figure 5:**
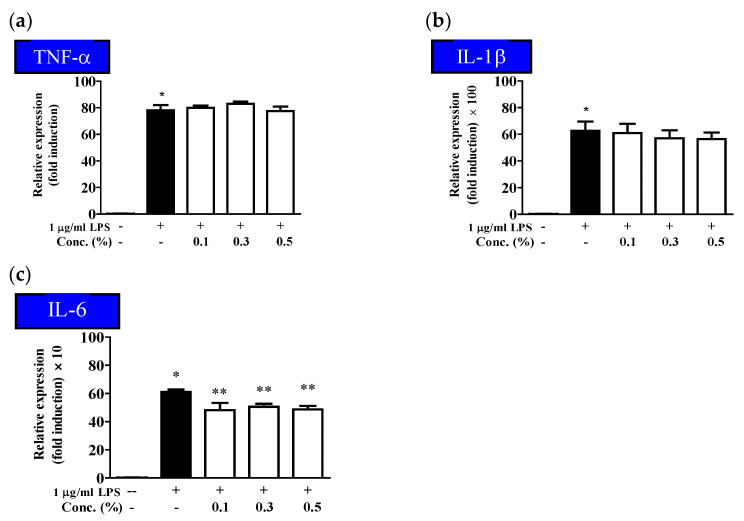
Inhibitory effect of EUFOC on the production of pro-inflammatory cytokines. (**a**) Tumor necrosis factor-α (TNFα), * *p* < 0.001 vs. negative control group. (**b**) Interleukin-1β (IL-1β), * *p* < 0.001 vs. negative control group. (**c**) Interleukin-6 (IL-6), * *p* < 0.001 vs. negative control group. ** *p* < 0.01 vs. LPS-treated group.

The inflammatory response is one of the immune system responses, and cytokines, such as TNF-α and IL-1β, are produced during the initial reaction, thereby increasing the activity of immune cells in tissue wounds or infection sites. Excessive secretion of cytokines involved in inflammatory reactions can induce acute or chronic inflammatory diseases [49,50]. To determine the effect of the EUFOC on the secretion of pro-inflammatory cytokines in HaCaT cells treated with LPS, the changes in the amounts of TNF-α, IL-1β, and IL-6 were measured after treatment with different concentrations of the EUFOC. As shown in Figure 5, the levels of all the cytokines were markedly increased by LPS treatment; however, IL-6 secretion was significantly suppressed by EUFOC treatments (Figure 5c) but not TNF-α and IL-1β (Figure 5a,b). TNF-α is a major endogenous mediator in the early stage of inflammatory response, while IL-1β is a pro-inflammatory cytokine produced during the inflammatory response and a major cytokine involved in the inflammatory response [51]. Therefore, our findings indicated that the EUFOC exhibited an anti-inflammatory effect by inhibiting the late stage of inflammatory response, but not the early stage and pro-inflammatory response.

### 2.5. Inhibitory Effect of the EUFOC on COX-2 and iNOS Expression

The inflammatory response is mediated by various pathways and is closely related to cyclooxygenase-2 (COX-2) and inducible nitric oxide synthase (iNOS) [15,52]. COX is an enzyme that converts arachidonic acid into prostaglandins and exists in two forms: COX-1 and COX-2. In particular, COX-2 is expressed in response to stimuli, such as cell growth factors, cytokines, tumor promoters, and reactive oxygen species, which cause inflammatory diseases [53,54,55]. iNOS generates NO to defend the body in response to external stimuli, and excessive production of NO results in inflammation, inducing tissue damage, genetic mutation, and neural damage [56].

**Figure 6:**
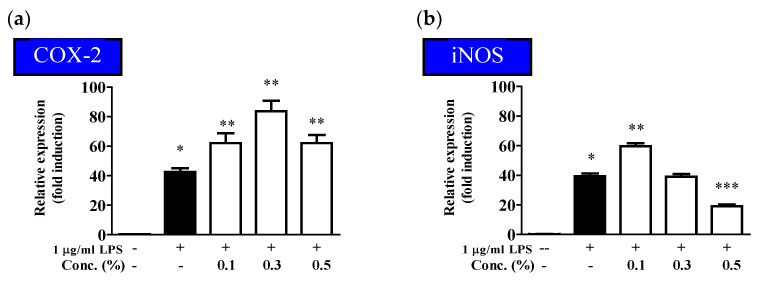
Inhibitory effects of EUFOC on mRNA expression of lipopolysaccharides (LPS)-induced pro-inflammatory cytokines. (**a**) Quantification of COX-2 expression. * *p* < 0.01 vs. negative control group; ** *p* < 0.05 vs. LPS-treated group. (**b**) Quantification of iNOS expression. * *p* < 0.01 vs. negative control group; ** *p* < 0.05 and *** *p* < 0.05 vs. LPS-treated group.

LPS-stimulated HaCaT cells were treated with the EUFOC to observe the expression of inflammatory mediators, such as COX-2 and iNOS. As shown in Figure 6, the protein expression of COX-2 and iNOS increased when HaCaT cells were treated with LPS, and the increased protein expression tended to decrease in a concentration-dependent manner after EUFOC treatment. These findings confirmed that the EUFOC inhibited the expression of the inflammatory mediators COX-2 and iNOS in HaCaT cells, indicating its potential as an anti-inflammatory agent.

In addition to immunoglobulins, which trigger inflammatory reactions in response to external factors, macrophages are also important for immunity [56]. Inflammatory reactions are triggered by LPS, an endotoxin, and anti-inflammatory experiments are conducted at the cellular stage [57]. RAW 264.7 cells were treated with 10 ng/mL of LPS to induce an inflammatory response and increase the expression of TNF-α and iNOS, which are inflammatory mediators. Next, the cells were treated with the EUFOC to confirm the degree of cytokine inhibition [57].

As indicated in Figure 6, the cells were treated with the EUFOC (0.1, 0.3, and 0.5% (*v*/*v*), and RNA was extracted from the treated cells. The mRNA levels of COX-2 and iNOS were compared using RT-PCR (Figure 6). COX-2 expression significantly increased in the LPS-treated group, and no suppression was observed in the EUFOC-treated groups (Figure 6a). Similarly, LPS treatment significantly increased iNOS mRNA expression. EUFOC treatment significantly reduced iNOS expression at relatively high concentrations (0.5%), but not at lower concentrations (Figure 6b). Although the EUFOC could not regulate the expression of COX-2, it exerted anti-inflammatory effects by regulating the expression of iNOS, which indicated its role in the inflammatory response.

Stimulus-induced iNOS produces a large amount of NO over a long period, and the generated NO is characterized by cytotoxicity to surrounding tissues and its ability to activate guanyl cyclase. Because NO is very small, reactive, and electrically neutral, it immediately spreads from the site of synthesis in all directions, intensifying inflammation by promoting the biosynthesis of inflammatory mediators as well as promoting inflammatory reactions, such as vascular permeability and edema [58,59].

### 2.6. HA Promotes Intracellular Synthesis

According to previous studies, some natural products are involved in the synthesis and maintenance of HA through various pathways [60]. HA is a glycosaminoglycan composed of D-glucuronic acid and N-acetyl-D-glucosamine, which cannot easily enter the cells due to its relatively large molecular weight [61]. In general, HA production naturally decreases with age, resulting in a decrease in water homeostasis in cells, and it may also be reduced by external physical and chemical factors. In animal cells, different genes, such as HAS1, HAS2, and HAS3, are involved in HA synthesis, and the presence of hyaluronidase, an enzyme that decomposes HA, affects the water content of individual cells [62].

The enzymes that produce HA in animal cells are HA synthases (HAS), and HAS1, HAS2, and HAS3 have been identified to date [63,64]. HAS2 is the main gene involved in HA synthesis in animal fibroblasts and keratinocytes [65]. To confirm the effect of the EUFOC on HAS2 gene expression in keratinocytes, the cells were treated with 0.1, 0.3, or 0.5% EUFOC in 70% ethanol. After 24 h, RT-PCR was performed, and gene bands were confirmed by electrophoresis. HAS2 expression was confirmed at all the concentrations tested. In the dermal layer of animals, HA synthesis is controlled by growth factors, such as transforming growth factor β, platelet-derived growth factor BB, fibroblast growth factor, and epidermal growth factor (EGF), and it is also known to be affected by female growth factors [63,66]. EGF induces HA synthesis by activating signal transducers and activators of transcription (STAT)-3 and by inducing HAS2 gene expression [66]. Through this experiment, we confirmed that HAS2 expression in keratinocytes treated with EUFOC increased at all the test concentrations.

**Figure 7:**
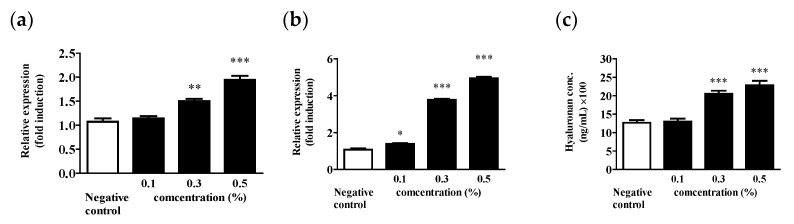
Enhancement effect of the EUFOC on HA synthesis. Confirmation of increased mRNA expression of (**a**) HAS2 and (**b**) HAS3. (**c**) Confirmation of increased expression of HA. * *p* < 0.05, ** *p* < 0.01, and *** *p* < 0.001 vs. experimental groups.

EUFOC treatment markedly increased the mRNA expression of HAS2 (Figure 7a) and HAS3 (Figure 7b) in HaCaT cells in a dose-dependent manner. These results suggest that EUFOC treatment can increase HA production and may play a role in the maintenance of water molecules in the dermal layer. Therefore, we measured changes in the HA contents in HcCaT cells after treatment with the EUFOC. The EUFOC induced HA production at a level similar to that of the control group at all the concentrations, indicating that it can maintain water molecules in the dermal layer (Figure 7c).

This study developed an EUFOC for the treatment and prevention of dermal inflammation. MTT and crystal red staining assays were conducted to evaluate the effect of the EUFOC on cell viability, and the results confirmed that no cytotoxicity occurred up to a concentration of 1.0%. In addition, cell viability was evaluated in 293T (human kidney) cells and HaCaT (human keratinocyte) cells, and cytotoxicity was not observed at 0.1, 0.3 and 0.5%. The evaluation of the inhibitory activity of the EUFOC on NO production revealed that it inhibited NO production at a concentration that did not inhibit cell viability. These results showed that LPS-induced NO production was reduced by the EUFOC, suggesting that the EUFOC is an effective anti-inflammatory agent. In order to confirm the effect of the EUFOC on the secretion of pro-inflammatory cytokines in LPS-treated HaCaT cells, the changes in the amounts of IL-1β and IL-6 induced by EUFOC treatment were measured. The LPS-induced increase in the secretion of all the cytokines was suppressed by the EUFOC in a dose-dependent manner. Moreover, the EUFOC effectively inhibited LPS-induced iNOS mRNA expression but not COX-2 expression in HaCaT cells. Although the EUFOC could not regulate the expression of COX-2, it exerted anti-inflammatory effects by regulating the expression of iNOS, suggesting its role in the inflammatory response. In addition, the EUFOC enhanced HA production at a level similar to that of the control group at all the test concentrations, indicating its potential to effectively maintain water molecules in the dermal layers. With regard to its safety on keratinocytes, our findings showed that there was no clear cytotoxicity up to 1.0%, and its effectiveness for HA synthesis was confirmed at different concentrations. As a possible limitation, these effects were observed after a relatively long incubation time with LPS, and we should verify these functional effects in a time-dependent manner. Furthermore, these functional effects can induce molecular remodeling such as the expression of all HAS families in various time courses; however, we must investigate this hypothesis in further studies with focused materials. The EUFOC, medicinal plant oil-based combined materials, showed significant ameliorative effects on LPS-induced inflammatory damages and provided evidence of tissue regeneration via transcriptional activation of hyaluronic acid synthesis. In conclusion, these results of the present study showed that the EUFOC, a natural oil manufactured using natural plant materials, is a potential medicinal and healthcare product with excellent anti-inflammatory and antioxidant properties.

## 3. Materials and Methods

### 3.1. Materials

The natural plant materials used in this study, namely, *Helianthus annus* seed, *Perilla frutescens*, *Prunus armeniaca*, *Paeonia lactiflora*, *Morus mongolica* (Bureau) C. K. Schneid bark, *Angelica gigas*, *Sophora flavescens*, *Gardenia jasminoides*, *Saururus chinensis*, *Houttuynia cordata*, *Acanthopanax cordata*, *Polygala tenuifolia*, and *Cimicifuga racemosa*, were purchased from Seoul Yangnyeongsi Market (Seoul, Republic of Korea). Dulbecco’s modified Eagle’s medium (DMEM), fetal bovine serum (FBS), penicillin-streptomycin (PS), and phosphate-buffered saline (PBS) were supplied by WelGENE (Daegu, Republic of Korea). Ham’s Nutrient Mixture F-12 (F-12) and trypsin-EDTA were obtained from Gibco-BRL (Rockville, MD, USA). Trypan blue was purchased from Sigma-Aldrich (St. Louis, MO, USA). TRIzol was obtained from Invitrogen (Carlsbad, CA, USA). Dimethyl sulfoxide (DMSO), MeOH, EtOH, chloroform, isopropanol, agarose, ethidium bromide (EtBr), and Tris were purchased from Bio-Rad (Hercules, CA, USA).

### 3.2. Composition of EUFOC

Essential oil components were extracted from the natural plant ingredients used in this study with an essential oil extractor (EssenLab-plus, Hanil Lab Tech, Yangju, Gyunggi-do, Republic of Korea). The essential oil obtained by mixing the essential oil extracts was named the EUFOC, and its composition is shown in Table 1. Unsaturated fatty acid content of the samples was 92.76%.

### 3.3. Cell Culture

All the cell lines were purchased from ATCC (Manassas, VA, USA) and cultured according to the manufacturer’s instructions. The cultured cells were washed thrice with cold phosphate-buffered saline (PBS) (0.1 M, pH 7.4). The cells were completely detached from the bottom of the culture dish using Trypsin-EDTA [57]. About 10 μL of cell suspension was added to 10 mL of culture medium supplemented with 10% FBS. The mixture was mixed with the same amount of trypan blue, and then about 10 μL was taken to count the number of cells using a hemocytometer at 200× magnification [57,67]. The number of cells was calculated by repeating the experiment 10 times and then calculating the average value. Each cell was cultured under 5% CO_2_ and full humidity at 37 °C. HaCaT cells were cultured in DMEM supplemented with 10% fetal bovine serum (FBS, Gibco) and 1% penicillin-streptomycin (PS, Gibco) under 5% CO_2_ at 37 °C. All cells were used in 4 to 5 passages from the first stock vial and then discarded.

### 3.4. Antioxidant Assessment

#### 3.4.1. Analysis of DPPH-Scavenging Ability

The antioxidant activity of the EUFOC was confirmed using a commercial DPPH free-radical-scavenging activity measurement method. First, DPPH was dissolved in ethanol to prepare a 0.1 mM DPPH solution, and the EUFOC was mixed with 0.1 mM DPPH solution at a ratio of 1:1 in a 96-well plate. The mixture of the sample and the DPPH solution was incubated at 37 °C for 30 min, and then the absorbance was measured at 517 nm. The DPPH-scavenging ability was calculated using the formula below:

DPPH free radical scavenging activity (%) = {1 − (A − B/C)} × 100

A: Absorbance after reacting sample with DPPH;

B: Absorbance after reacting sample and ethanol;

C: Absorbance after reacting ethanol and DPPH (blank).

#### 3.4.2. Analysis of SO-Scavenging Ability

The SO-scavenging ability of the EUFOC was confirmed using a commercial superoxide dismutase determination kit (Sigma-Aldrich, St. Louis, MO, USA). The samples were mixed with the reaction solution in the kit, placed in a 96-well plate, and incubated at 37 °C for 20 min. After measuring the absorbance at 450 nm, the SO-scavenging ability was calculated using the following formula:

SO-scavenging activity (%) = {[(C − D) − (A − B)]/(C − D)} × 100

A: Absorbance after incubating the sample with the reaction solution;

B: Absorbance after incubating the sample with the WST Working Solution;

C: Absorbance of the reaction solution;

D: Absorbance of the WST Working Solution.

### 3.5. Cell Viability Measurement Using MTT Assay

Abcam’s Crystal Violet Assay Kit (cell viability) (ab232855) and MTT reagents were used for cell viability assay. RAW 264.7 cells (5 × 10^4^ cells/well) were seeded into 96-well plates and left to stabilize in a 37 °C incubator for 24 h, followed by treatment with the EUFOC (0.05, 0.1, 0.3, 0.5, 1.0, or 2.0%).

The MTT solution was added after 24 h to confirm cytotoxicity. The sample was removed after 2 h and 150 μL of DMSO was added to each well to dissolve the formed formazan, followed by the measurement of absorbance at 540 nm.

MTT was used to confirm the cytotoxicity of the EUFOC in keratinocytes. HaCaT cells were cultured in DMEM supplemented with 10% fetal bovine serum (FBS, Gibco, Grand Island, NY, USA) and 1% penicillin-streptomycin (PS, Gibco, Grand Island, NY, USA) under 5% CO_2_ at 37 °C. The cells were seeded in 96-well plates at a density of 2 × 10^4^/well and then stabilized for 24 h. The EUFOC, diluted in serum-free medium, was further diluted with ethanol (70%) to prepare different concentrations of the sample. After 24 h, the medium was removed from the cells and 20 µL of MTT (5 mg/mL) was added, followed by incubation in a cell incubator (37 °C, 5% CO_2_) for 2 h. Next, MTT was removed, and 100 μL of DMSO was added. After ensuring that the crystals were dissolved in the stirrer, the absorbance was measured at 540 nm.

### 3.6. Identification of Inflammatory Genes Using RT-PCR

Macrophages respond to pathogens and cause an inflammatory response, producing pro-inflammatory cytokines such as TNF-α, IL-6, and IL-1β. In addition, inflammatory factors such as NO produced by iNOS and PGE2 produced by COX-2 are also secreted [68,69].

For inflammatory gene analysis, RAW 264.7 cells were seeded in 35 mm culture dish at a concentration of 3 × 10^5^ cells/well. The cells were treated with 10 ng/mL LPS and 0.05, 0.1, 0.3, 0.5, 1.0, or 2.0% EUFOC. After 24 h, the medium was removed, and RNA was isolated using TRIzol reagent. After RNA isolation, the samples were dried and re-dissolved in diethylpyrocarbonate-treated water. The isolated RNA was quantitated by absorbance measurement at 260 nm, and A260/A280 that ranged from 1.7 to 2.0. cDNAs was synthesized using a Bio-Rad iScript cDNA Synthesis Kit (Hercules, CA, USA). PCR conditions were as follows: 94 °C 15 min, 32–35 cycles, 94 °C 30 s, 50 °C 30 s, 72 °C 60 s, and 72 °C 10 min. The primers used were as follows: 5′-GGAGAGACTATCAAGATAGT-3′ (sense) and 5′-ATGGTCAGTAGACTTTTACA-3′ (antisense) for COX-2; 5′-GAGCGAGTTGTGGATTGTC-3′ (sense); 5′-CTCCTTTGAGCCCTTTGT-3′ (antisense) for iNOS; and 5′-CTGTCCCTGTATGCCTCTG-3′ (sense) and 5′-ATGTCACGCACGATTTCC-30 (antisense) for β-actin [70].

### 3.7. Inhibition of NO Production

NO production was measured using the Griess method. RAW 264.7 cells, pre-cultured in DMEM growth medium supplemented with 10% FBS, were seeded in 24-well tissue culture plates at a concentration of 5 × 10^4^ cells/well and cultured for 1 day in a 5% CO_2_ incubator. After removing the medium and starving the cells in a serum-free medium for 12 h, the EUFOC was added at the required concentration, followed by incubation for 30 min. Next, 10 ng/mL LPS (Sigma-Aldrich, St. Louis, MO, USA) was added, followed by culturing for 18 h. After incubation, the supernatant was collected and transferred to 96-well plates; Griess reagent (Sigma-Aldrich, St. Louis, MO, USA) was added, followed by incubation at room temperature for 15 min. The absorbance was measured at 540 nm using an enzyme-linked immunosorbent assay (ELISA) reader.

### 3.8. ELISA Test

The cells were seeded in 6-well plates at a concentration of 2 × 10^5^/mL. After 24 h, the cells were washed twice with serum-free DMEM. The cells were re-cultured in serum-free DMEM containing 1% DMSO. After 24 h, 350 μL of medium was removed, and after another 24 h, the same amount was removed. After centrifugation at 15,000× *g* for 5 min, the supernatant was removed and stored at −20 °C until ELISA. An HA-ELISA kit (Echelon, Salt Lake City, UT, USA) was used to measure the HA produced by the EUFOC in keratinocytes. The 48 h treatment increased HA production in all the samples, compared with the 24 h treatment. Therefore, the EUFOC, at an appropriate concentration, can enhance HA production in keratinocytes. Further studies are necessary to explore the mechanisms of action and determine the optimal concentration of the EUFOC. All-trans retinoic acid was used as a positive control.

### 3.9. Quantative PCR

Keratinocytes were seeded into 6-well plates at 2 × 10^5^/well and then stabilized for 24 h. The medium was replaced with serum-free DMEM and the EUFOC was diluted with 70% ethanol. The final concentration of DMSO was adjusted to 0.1% in all the experimental groups, and 24 h after sample treatment, RNA was extracted using easy-BLUE™ Total RNA Extraction Kit (iNtRON). After measuring the RNA purity and concentration, 1 μg of RNA extracted from each 6-well plate was used to synthesize cDNAs using a Power cDNA Synthesis Kit (iNtRON). DNA was amplified using SYBR Green Master Mix (Bio-Rad, CA, USA) in an ABI Prism 7900HT sequence-detection system (Applied Biosystems, Lennik, Belgium). Primers to HAS-2 (CTGGGACGAAGTGTGGATTATG and GATGAGGCTGGGTCAAGCAT) and HAS-3 (GCCCTCGGCGATTCG and TGGATCCAGCACAGTGTCAGA) were used at 300 nM [71]. Results were expressed relative to the number of beta-actin transcripts used as an internal control.

### 3.10. Promotion of HA Production

We evaluated the ability of the EUFOC to promote HA production in HaCaT cells using the ELISA method. Different concentrations of the EUFOC (0.1%, 0.3%, and 0.5%) were prepared with the cell medium. HaCaT cells pre-cultured in a DMEM growth medium supplemented with 10% FBS were added to 96-well plates at a concentration of 2 × 10^4^ cells/well and cultured in a 5% CO_2_ incubator for 24 h. The medium was removed and the EUFOC diluted in serum-free medium was added, followed by incubation for 24 h. After incubation, the supernatant was collected and subjected to ELISA using the HA-ELISA kit (DHYAL0, R&D systems, Minneapolis, MN, USA).

### 3.11. Statistical Analysis

The experiment conducted in this study was repeated seven times or more in total, and the statistical significance test between the data of the experimental groups was performed using paired samples t-tests (GraphPad Prism 5). The results are expressed as mean ± standard deviation. Statistical significance is indicated as * *p* < 0.05, ** *p* value ≤ 0.01.

## Figures and Tables

**Table 1:** Composition ratio of the EUFOC.

NCI	CAS No	Contents (%)
*Helianthus annuus* seed oil	8001-21-6	Add to 100%
*Perilla frutrscens* oil	68132-21-8	20%
*Prunus armeniaca* oil	72869-69-3	3%
*Paeonia lactiflora* extract	7732-18-5	1%
*Cimicifuga racemosa* root extract	84776-26-1	1%
*Gardenia jasminoides* fruit extract	92457-01-7	1%
*Morus mongolica* (Bureau) C. K. schneid extract	94167-05-2	0.8%
*Houttuynia cordata* extract	164288-50-0	0.8%
*Saururus chinensis* extract	91770-72-8	0.8%
*Angelica gigas* root extract	84775-41-7	0.8%
*Centella Asiatica* extract	84776-24-9	0.5%
*Polygala tenuifolia* root extract	94167-09-6	0.5%
*Sophora flavescens* extract	519-02-8	0.5%
*Acanthopanax cordata* extract	39432-56-9	0.5%

## Data Availability

The data presented in this study are available.

## Supplementary Materials

The following supporting information can be downloaded at: https://www.mdpi.com/article/10.3390/molecules28083554/s1, Introduction S1: Supplementary introduction.

## Sample Availability

Samples of the compounds: EUFOC are available from the Eouidang Agricultural Company.
