# Supplementary material for: Unsaturated Fatty Acids Complex Regulates Inflammatory Cytokine Production through the Hyaluronic Acid Pathway
Source: Molecules. 2023 Apr 18;28(8):3554. doi: 10.3390/molecules28083554 (PMC10142694; doi:10.3390/molecules28083554)
Supplement: Supplementary file 1 [file molecules-28-03554-s001.zip › molecules-2245719-supplementary.pdf]

Supplementary Introduction S1

Unsaturated Fatty Acids Complex Regulates Inflammatory Cytokine Production through Hyaluronic Acid Pathway

Gi-Beum Kim [1,†], Kwansung Seo [2,†], Jong-Ung Youn [3], Il Keun Kwon [2], Jinny Park [4], Kwang-Hyun Park [5,‡,*] and Jong-Suk Kim [1,*]

*Paeonia lactiflor*a is a perennial plant in the genus Paeonia of the family *Paeoniaceae* of *Dicotyledoneae*, native to Korea. Flowers are mainly used for gardening, and roots are used as therapeutic medicines for pain, abdominal pain, menstrual pain, amenorrhea, hematemesis, anemia, and bruises, etc. [1]. *Paeonia lactiflora* extract is used as a functional pharmaceuticals, and the ingredients obtained by extracting the flowers of the Chinese peony are used.

Morus mongolica (Bureau) C. K.Schneid is a medicinal ingredient made from the root bark of from the white mulberry in the family Moraceae or plants of the same genus [2,3]. It treats chronic cough and asthma caused by waste heat and has a diuretic effect. It is also used for acute pyelitis, frailty edema, blood pressure drops, hepatitis epidemic, nosebleeds, and hemoptysis [4,5]. Pharmaceutical actions including cough, diuretic, blood pressure drop, sedation, analgesic, antipyretic, antispasmodic, and antibacterial action, etc. have been reported [5]. *Morus mongolica (Bureau) C. K.Schneid* extract is a medicinal ingredient commonly used in whitening pharmaceautical medicines. The white bark of the mulberry root is called

Angelica gigas is a dried root of Angelica, perennial grass belonging to the Apiaceae family [6]. *Angelica gigas* promotes blood production, so it is effective in the prevention and treatment of anemia, menstrual irregularity caused by stress, and is also good for precious blood and women's diseases. It also smoothest stool and stops hemorrhage [7]. In pharmacological experiments, sedation, analgesic, antibacterial action, and diuretic action were found. It is used for haemorrhage, menstrual irregularity, amenorrhea, hematemesis, anemia, bruises, postpartum abdominal pain, bruises, constipation, dysentery, rhinitis, etc., and is also used for uterine hypoplasia, neurasthenia, and uterine bleeding, etc. [8]. *Angelica gigas* root Extract is a water-soluble natural medicinal and healthcare ingredient extracted from the dried root of *Angelica gigas* as a raw material. *Chinese Angelica* root extract improves blood circulation and relieves extravasated blood, so it can be used in medicine for extracellular secretion control, nutrient supplementation, and dark circle relief, etc. It also acts on the proliferation of vascular epithelial cells, helping to smooth blood circulation and protect capillaries. It has an anti-aging effect on the activation rate of luciferase and the proliferation of hepatocytes.

Sophora flavescens is perennial grass in the order Rosales of the family *Fabaceae* of *dicotyledoneae*, and in Korean oriental medicine, dried roots are used as a medicine for indigestion, neuralgia, hepatitis, tonsillitis, pneumonia, and jaundice. Anti-inflammatory, immunity action, antibacterial action, leukocyte-increasing effect, etc. have been reported. *Sophora flavescens* extract is effective in maintenance of wate, promoting hair growth, promoting blood circulation, and antibacterial action, etc. It prevents acne and dermatitis to keep the clean condition. It also relieves dandruff and scalp itching [9].

Gardenia jasminoides is a broadleaf evergreen shrub belonging to the Rubiaceae family, and its fruits have antipyretic, diarrhea, hemostasis, and anti-inflammatory effects, so it is used as medicine in Korean oriental medicine. It is used for symptoms such as hepatitis, jaundice, and hematemesis [10]. *Gardenia jasminoides* fruit extract, which is used as a medicinal and healthcare raw material of Rapern, is an ingredient extracted from the fruit of the gardenia tree. In Korea, *Gardenia jasminoides* was introduced from China 500 years ago. *Gardenia jasminoides* is also used as one of the yellow vegetable

dyes, which is slightly redder than the yellow of turmeric, and is often used as an ornamental tree because of its fragrant flowers and beautiful fruits.

Saururus chinensis is perennial grass from Jeju Island and some areas of Jirisan Mountain. Since ancient times, it has been used in the treatment of detoxification, dispersing swelling, hepatitis, and jaundice, and is known to reduce heart-related diseases or lower high blood pressure in Korean oriental medicine due to its action to clear blood and blood vessels [11,12]. In particular, *Saururus chinensis* contains amino acids, organic acids, sugars, and tannins, so it is effective in purulent mastitis, urethral pain after urinating, geriatric diseases, and hypertension, etc. [12].

Houttuynia cordata is also called Heartleaf Houttuynia, and is perennial grass belonging to the Saururaceae family. The plant body before flowering is used as a diuretic and anthelmintic, and among the common people, it is used for a running sore, suppuration, and hemorrhoids, and in Korean oriental medicine, it is used for gonorrhea, enteritis, urinary tract infections, pneumonia, and bronchitis [13].

Acanthopanax cordata extract is extracted from the roots of *Eleutherococcus senticosus*, and it is a plant that can live in extreme regions. Glycosides such as Eleutherosid A, B, B1, B2, B3, B4, C, D, E, F, G, etc. are distributed in the roots of *Eleutherococcus Senticosus*, helping improve immunity along with physiological activities such as anticancer, antioxidant, and anti-mutagenic effects [14].

Cimicifuga racemosa refers to a medicinal ingredient made from the rhizome of *Cimicifuga racemosa* in the Ranunculaceae family or plants belonging to the same genus, and it is used for an extrinsic fever, headache, early measles rash, headache caused by fever poisoning, sore throat, gum diseases, and tongue diseases, malaise caused by physical weakness, prolonged diarrhea, prolapse, terrine effusion, etc. [15]. *Cimicifuga racemosa* root extract is involved in excellently inhibiting the isolation of histamine, a main factor of allergy, and helps to relieve allergies.

Helianthus annus seeds contain about 50% of fatty oil, and linoleic acid and phospholipids, which account for 70% of them, are effective in inhibiting thrombus of hyperlipidemia and hyper cholesterol, as well as anticancer, immune regulation, and involvement in lipid metabolism [16]. *Helianthus Annuus* (Sunflower) Seed Oil is an oil extracted from sunflower seeds. Sunflower seed oil is rich in nutrients that are good for the body, such as protein, unsaturated fatty acids such as linoleic acid and oleic acid, lecithin, carotenoids, tocopherol, vitamins A, C, D, and dietary fiber. It has a warm nature, so it is very good for people with weak digestion and stomach problems when ingested. When you feel bloated or indigestible, take a spoonful at a time for better digestion. It inhibits the growth of Helicobacter pylori and also prevents gastric ulcers and gastric cancer. In addition, it is good for cardiovascular diseases because it is linoleic acid-rich oil compounds that maintains integrity of blood vessels. It has a structure similar to that of a newborn's vernix caseosa, so rubbing it on newborns and infants protects from infection of harmful bacteria. Sunflower seed oil contains very high vitamin E. Vitamin E is an antioxidant that prevents damage to the dermal layers due to harmful substances such as UV rays and relieves scars and wrinkles, making the healthy dermal environments. It also contains abundant β-carotene, which is converted into vitamin A, an antioxidant, to protect the integument system from UV rays and prevent problems such as sunburn and skin cancer.

Perilla frutrscens oil has a high content of linolenic acid and has anti-atherosclerosis, anti-hypertensive, improvement of the cognitive ability, and other functions. *Perilla frutrscens* oil is a fixed oil (non-volatile oil) component obtained from perilla seeds. Perilla seeds (kernel) contain glycerides of oleic acid and linoleic acid as fatty oils [17].

Prunus armeniaca oil contains abundant minerals and vitamins and is known to be particularly effective in relieving dermalgia with vitamin A-rich oil [18]. Also, the content of oleic acid and linolenic acid is high. *Prunus armeniaca* oil is an oil extracted from Apricot seeds, which are called *Armeniacae Semen*, and it is high in phosphorus, protein and fat and also contains amygdalin. *Prunus armeniaca* oil contains oleic acid, linoleic acid, and vitamin E, and maintains a transparent liquid state even at low temperatures because the proportion of saturated fatty acids is small. Therefore, in cold climates, *Prunus armeniaca* oil is widely used as a body oil. With its continuously emollient activity, it is effective in improving sun-burn by helping the blood circulation as it is quickly absorbed into the dermal layers and accelerates removing necrosis cells. Additionally, *Prunus armeniaca* oil is the most similar oil to sweet almond oil and can be applied to lip balm, cream, hair care, and natural soap.

Supplementary References

1. Li, P.; Shen, J.; Wang, Z.; Liu, S.; Liu, Q.; Li, Y.; He, C.; Xiao, P. Genus Paeonia: A comprehensive review on traditional uses, phytochemistry, pharmacological activities, clinical application, and toxicology. *J. Ethnopharmacol* **2021**, *269*, 113708.
2. Dawid-Pać, R. Medicinal plants used in treatment of inflammatory skin diseases. *Postepy Dermatol Alergol* 2013, *3*, 170–177.
3. Armenova, N.; Tsigoriyna, L.; Arsov, A.; Petrov, K.; Petrova, P. Microbial Detoxification of Residual Pesticides in Fermented Foods: Current Status and Prospects. *Foods* 2023, *12*, 1163.
4. Kim, H.; Song, M.-J. Analysis and recordings of orally transmitted knowledge about medicinal plants in the southern mountainous region of Korea. *J. Ethnopharmacol* **2011**, *134*, 676–696.
5. Chang, B.-Y.; Koo, B.-S.; Kim, S.-Y. Pharmacological Activities for Morus alba L., Focusing on the Immunostimulatory Property from the Fruit Aqueous Extract. *Foods* **2021**, *10*, 1966.
6. Avula, B.; Joshi, V.; Reddy V. L, N.; Choi, Y.-W.; Khan, I. Simultaneous Determination of Eight Coumarins in Angelica gigas and in Various Other Angelica Species by High Performance Liquid Chromatography and Comparative Micro-Morphology Study of Angelica Species. Planta Med **2007**, *73*, 1509–1516.
7. Kim, M.-C.; Lee, G.-H.; Kim, S.-J.; Chung, W.-S.; Kim, S.-S.; Ko, S.-G.; Um, J.-Y. Immune-enhancing effect of Danggwibohyeoltang, an extract from *Astragali Radix*and*Angelicae gigantis Radix*,*in vitro* and *in vivo*. *Immunopharmacol. Immunotoxicol* 2011, *34*, 66–73.
8. Batiha, G. E.-S.; Shaheen, H. M.; Elhawary, E. A.; Mostafa, N. M.; Eldahshan, O. A.; Sabatier, J.-M. Phytochemical Constituents, Folk Medicinal Uses, and Biological Activities of Genus Angelica: A Review. *Molecules* **2022**, *28*, 267.
9. I E, C. The safe usage of herbal medicines: counterindications, cross-reactivity and toxicity. *Pharmacogn Commn*. **2014**, *5*, 2–50.
10. Miwa, T. Study on gardenia florida l. (fructus gardeniae) as a remedy for icterus. *Japanese Journal of Pharmacology* **1955**, *4*, 69–81.
11. Kim, J.-H.; An, C.; Hwang, S. D.; Kim, Y. S. Ceriporia lacerata Mycelium Culture Medium as a Novel Anti-Aging Microbial Material for Cosmeceutical Application. *Cosmetics* 2021, *8*, 101.
12. Kang, C.-S., Lee, M.-J., Park, C.-B., Bang, I. S. Study on the Antioxidative and Physiological Activities of Saururus chinensis Extract. *J. Life Sci* **2012**, 22, 807–814.
13. Ghosh, A.; Ghosh, B.; Parihar, N.; Ilaweibaphyrnai, M.; Panda, S. R.; Alexander, A.; Chella, N.; Murty, U.; Naidu, V.; Kumar G, J.; Pemmaraju, D. B. Nutraceutical prospects of Houttuynia cordata against the infectious viruses. *Food Biosci* **2022**, *50*, 101977.
14. Kim, Y.-H.; Cho, M.; Kim, D.-B.; Shin, G.-H.; Lee, J.-H.; Lee, J.; Park, S.-O.; Lee, S.-J.; Shin, H.; Lee, O.-H. The Antioxidant Activity and Their Major Antioxidant Compounds from Acanthopanax senticosus and A. koreanum. *Molecules* **2015**, *20*, 13281–13295.
15. Ulbricht, C.; Windsor, R. C. An Evidence-Based Systematic Review of Black cohosh (*Cimicifuga racemosa,Actaea racemosa*) by the Natural Standard Research Collaboration. *J. Diet. Suppl* **2014**, *12*, 265–358.
16. Choi, Y.-S.; Park, K.-S.; Kim, H.-W.; Hwang, K.-E.; Song, D.-H.; Choi, M.-S.; Lee, S.-Y.; Paik, H.-D.; Kim, C.-J. Quality characteristics of reduced-fat frankfurters with pork fat replaced by sunflower seed oils and dietary fiber extracted from makgeolli lees. *Meat Sci* 2013, *93*, 652–658.
17. Dhyani, A.; Chopra, R.; Garg, M. A Review on Nutritional Value, Functional Properties and Pharmacological Application of Perilla (Perilla Frutescens L.). *Biomed. Pharmacol. J* **2019**, *12*, 649–660.
18. MATTHÄUS, B.; ÖZCAN, M. M. Fatty acids and tocopherol contents of some*prunus*spp. kernel oils. *J. Food Lipids* **2009**, *16*, 187–199.
